# Cardiac Magnetic Resonance in Stable Coronary Artery Disease: Added Prognostic Value to Conventional Risk Profiling

**DOI:** 10.1155/2018/2806148

**Published:** 2018-06-21

**Authors:** Oronzo Catalano, Guido Moro, Alessia Mori, Mariarosa Perotti, Alessandra Gualco, Mauro Frascaroli, Clara Pesarin, Carlo Napolitano, Ntobeko A. B. Ntusi, Silvia G. Priori

**Affiliations:** ^1^Division of Cardiology, Istituti Clinici Scientifici Maugeri, Pavia, Italy; ^2^Division of Radiology, Istituti Clinici Scientifici Maugeri, Pavia, Italy; ^3^Occupational Medicine Unit, Istituti Clinici Scientifici Maugeri, Pavia, Italy; ^4^Molecular Cardiology, Istituti Clinici Scientifici Maugeri, Pavia, Italy; ^5^Department of Medicine, Cape Universities Body Imaging Centre, University of Cape Town and Groote Schuur Hospital, Cape Town, South Africa; ^6^Division of Cardiology, Molecular Cardiology, Istituti Clinici Scientifici Maugeri, Pavia, Italy; ^7^University of Pavia, Pavia, Italy

## Abstract

**Aims:**

Cardiovascular magnetic resonance** (**CMR) permits a comprehensive evaluation of stable coronary artery disease (CAD). We sought to assess whether, in a large contemporaneous population receiving optimal medical therapy, CMR independently predicts prognosis beyond conventional cardiovascular risk factors (RF).

**Methods:**

We performed a single centre, observational prospective study that enrolled 465 CAD patients (80% males; 63±11 years), optimally treated with ACE-inhibitors/ARB, aspirin, and statins (76-85%). Assessments included conventional evaluation (clinical history, atherosclerosis RF, electrocardiography, and echocardiography) and a comprehensive CMR with LV dimensions/function, late gadolinium enhancement (LGE), and stress perfusion CMR (SPCMR).

**Results:**

During a median follow-up of 62 months (IQR 23-74) there were 50 deaths and 92 major adverse cardiovascular events (MACE). CMR variables improved multivariate model prediction power of mortality and MACE over traditional RF alone (F-test p<0.05 and p<0.001, respectively). LGE was an independent prognostic factor of mortality (hazard ratio [95% CI]: 3.4 [1.3−8.8]); moreover, LGE (3.3 [1.7−6.3]) and SPCMR (2.1 [1.4−3.2]) were the best predictors of MACE.

**Conclusion:**

LGE is an independent noninvasive marker of mortality in the long term in patients with stable CAD and optimized medical therapy. Furthermore, LGE and SPCMR independently predict MACE beyond conventional risk stratification.

## 1. Introduction

Coronary artery disease (CAD) is a leading cause of mortality worldwide [1]. In spite of improvements in medical therapy and revascularization procedures over the last few decades, many areas of uncertainty still exist. In patients with stable CAD, it is unclear why, despite prevalent use of stress imaging for inducible ischemia through techniques with recognized prognostic value, including stress echocardiography and single photon emission computerized tomography [2, 3], myocardial revascularization often fails to reduce hard endpoints, even in patients with extensive 3-vessel CAD, when compared to optimal medical therapy [4–8].

Cardiovascular magnetic resonance (CMR) is an established, robust, noninvasive, and radiation-free imaging technique for assessing CAD [9]. It allows in a single examination simultaneous evaluation of myocardial contractility, mass, wall motion, perfusion, tissue characteristics, and viability. In particular, dobutamine stress CMR and stress perfusion CMR (SPCMR) can assess the hemodynamic significance of intermediate coronary stenoses and late gadolinium enhancement (LGE) CMR is useful for imaging focal myocardial scar determining viability [10]. However, it is unclear how a comprehensive CMR assessment strategy may impact long-term outcomes in a contemporaneous cohort of patients with stable CAD who are optimally treated. Specifically, there is lack of clarity as to whether such a CMR strategy may improve risk stratification beyond well-known conventional risk factors.

Therefore, the aim of our study was twofold: (1) to assess whether, in a large, well-characterized population with optimal medical therapy undergoing CMR for stable CAD, the presence of left ventricle (LV) dysfunction, SPCMR abnormalities, and/or fibrosis (assessed by LGE) was independent predictor of all-cause mortality in the long term, independently of traditional cardiovascular risk factors and (2) to assess the prognostic impact of CMR on major adverse cardiovascular events (MACE).

## 2. Methods

### 2.1. Study Population and Design

**Figure 1:**
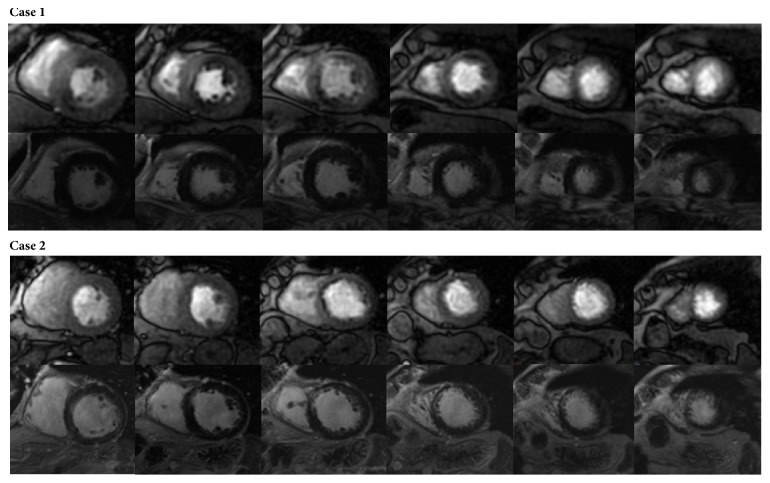
*First pass dipyridamole stress perfusion and late gadolinium enhancement CMR assessment*. Case 1 shows a positive stress perfusion study in two coronary territories by reason of a basal-mid anterior (partially) transmural as well as of a basal inferoseptal and mid inferior subendocardial stress perfusion defects (upper images) without late gadolinium enhancement (lower images). Case 2 depicts a negative stress perfusion study because of a mid anteroseptal and inferoseptal subendocardial stress perfusion defect (upper images) matching a similar late gadolinium enhancement area (lower images).

We performed a single centre, observational prospective study. Inclusion criteria: consecutive patients referred clinically for CMR with either definite diagnosis or a history suggesting stable CAD were enrolled. Exclusion criteria: we excluded patients with recent acute coronary syndrome (within 6 weeks), previous hospitalization for heart failure (NYHA class IV or need of infusive therapy) and signs of myocarditis, infiltrative or hypertrophic cardiomyopathy, and pericardial disease. Part of the study cohort participated in an earlier study on independent prognostic value of LGE [11] in which SPCMR assessment was not considered. Clinical history collection, electrocardiogram, and echocardiography evaluation criteria and CMR protocol (except for SPCMR) were the same as in the preceding study. In summary, patients underwent a conventional clinical and instrumental assessment as well as a comprehensive CMR evaluation. First pass SPCMR was evaluated 7.5 minutes after an intravenous infusion of dipyridamole stress (0.56 mg/kg for 4 minutes). Six contiguous short axis images, covering most of LV, were acquired. After stress perfusion images acquisition patients received an injection of aminophylline 240 mg i.v. in 10 minutes to antagonize dipyridamole effects. Perfusion images were semiquantitatively evaluated by two blinded operators with more than ten-year CMR experience and slice-to-slice compared with the corresponding LGE images. In each of the 17-segment standard segmentation, a perfusion defect was considered significant if it involved ≥75% of myocardium wall thickness, persisted at least 3 frames, and was detected in absence of LGE. A SPCMR study was considered positive if at least 2 segments (that equals to more than 10% of segments) showed significant perfusion defect.  Figure 1 shows two studies with perfusion defects and, respectively, absence (positive SPCMR) or presence of LGE (negative SPCMR).

Informed consent to participate in the research study was obtained from each patient and the study protocol conforms to the ethical guidelines of the 1975 Declaration of Helsinki as reflected in a priori approval by the Institutional Review Board of the Fondazione Salvatore Maugeri (Pavia, Italy).

### 2.2. Follow-Up

Follow-up visits were conducted at our centre every 1–24 months, depending on clinical severity. Telephonic follow-up was performed for those patients whose last visit date was 6 months prior to the database closure. The primary outcome measure was all-cause mortality. The secondary outcome measure was a composite clinical endpoint of MACE, including all-cause mortality and hospitalization due to new onset New York Heart Association (NYHA) class IV or needing intravenous diuretics for heart failure, acute coronary syndrome (ACS), or myocardial revascularization procedures. Revascularization occurring within one month of CMR imaging was considered as CMR related and was not calculated as a separate MACE. Cases with more than one MACE were censored at the time of the first event.

### 2.3. Statistics

Categorical variables were expressed as counts and percentage, continuous variables as mean ± standard deviation or interquartile range (IQR). Two-sided P<0.05 was the significance level for hypothesis testing and SPSS Statistics 18.0 (IBM, USA) was the statistical package used. Differences at baseline between patients with and without events were tested with Pearson *χ*^2^ or Fisher's exact test for categorical variables and Student's* t*-test or Mann–Whitney U test for continuous variables, where appropriate. Univariate hazard ratios were calculated by Cox proportional hazard analysis after converting continuous and ordinal variables into dichotomous variables. Threshold values were taken from the literature or were set equal to the 95th percentiles of the entire study population, when established threshold values were lacking (for example LGE cut-off was 40% of LV mass). Threshold values are indicated in brackets after each nondichotomous variable in Table 2. Proportional hazard assumption was graphically tested using plots of the log estimated cumulative baseline hazard against time. Conventional variables correlating with prognosis (p<0.1) at multivariate analysis (stepwise forward selection, forceful introduction of LVEF) were used to build the final model in which CMR assessment was introduced at the last step to test the hypothesis of its additional prognostic value over total mortality or MACE, on top of a conventional risk stratification approach. F-test for extra sum of square principle was applied to assess goodness of fit of the final model with respect to the conventional nested model.

## 3. Results

**Figure 2:**
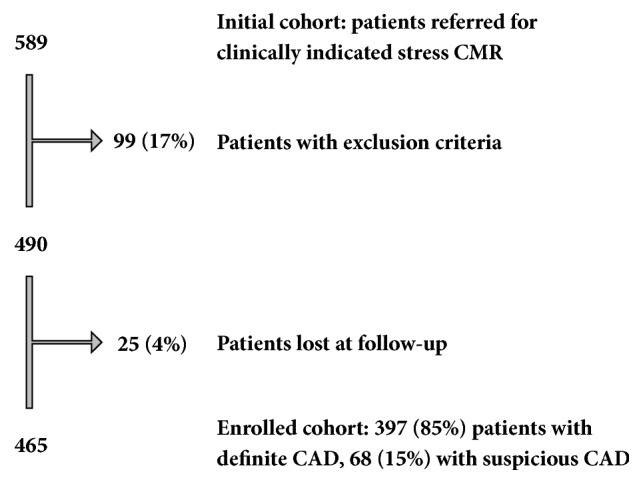
*Study enrollment flow chart*. Final cohort enrolled in the study after considering exclusion criteria and patients lost at follow-up.

Five hundred eighty-nine patients were referred to our unit for CMR assessment during the period of interest. Forty-five (8%) were excluded as they presented exclusion criteria, and 54 (9%) because stress perfusion was not performed for clinical reason. Twenty-five (4%) patients were lost to follow-up. Thus, 465 patients entered the study, 397 (85%) with a definite diagnosis of CAD at enrollment and 68 (15%) with a history of likely CAD (Figure 2 summarizes the study flow chart). Patients were followed up for a median follow-up time of 62 months (interquartile range: 23-74), during which 142 events occurred (50 deaths, 20 new onset heart failure cases, 16 ACS, and 56 myocardial revascularization procedures). Twelve cases had more than one MACE.

Main baseline characteristics are reported in Table 1. Overall the study cohort was characterized by middle aged patients (63 years ± 11), with prevalence of male sex (80%) and preserved LV systolic function (LVEF at ECHO 53% ± 13%). History of previous myocardial infarction was elicited in two-thirds, LM/3−vessel CAD in one-third, and diabetes in one-fifth of cases. Pharmacological treatment was characterized by optimal use of angiotensin converting enzyme- (ACE-) inhibitors/angiotensin receptor blockers (ARB), aspirin, and statins (76-85%). Accordingly, at the last follow-up contact, (1) mean LDL cholesterol value was 99 ± 32 mg/dl and LDL was < 100 mg/dl in 63% of cases, (2) mean systolic blood pressure (SBP) was 115 ± 16 mmHg and SBP was < 140 mmHg in 95% of cases and (3) mean diastolic blood pressure (DBP) was 70 ± 8 mmHg, and DBP was < 90 mmHg in 100% of cases. A fifth of patients had a positive dipyridamole SPCMR at baseline and a quarter of patients underwent at least one revascularization procedure during the follow-up.

### 3.1. Risk Stratification by Conventional Assessment

#### 3.1.1. Prediction of All-Cause Mortality

Univariate analyses showed, in keeping with published literature, an association between all-cause mortality and LVEF and several other predefined factors. Among them, only a revascularization procedure after the study enrollment was a protecting factor from mortality. Univariate hazard ratios are shown in Table 2.

Stepwise inclusion of variables reaching the predefined univariate significance value threshold (p<0.1) into a multivariate Cox model in which LVEF was included at the first step significantly improved the model predictability (extra sum of square *χ*^2^ 97 versus 69, F-test: p<0.001) with respect to considering LVEF alone. However, only ACS in the follow-up (Hazard Ratio [95% C.I]: 9.1 [3.8–21.8]; p<0.001), LV mass (2.6 [1.1−5.9]; p<0.05), QTc interval (2.6 [1.3−5.1]; p<0.01), and heart rate (2.4 [1.2−4.8]; p<0.05) were independently associated with an adverse prognosis after entering LVEF (3.9 [1.8−8.3]; p<0.001).

#### 3.1.2. Prediction of MACE

Univariate analyses identified many conventional variables associated with MACE. Indeed, all variables associated with all-cause mortality, except mitral regurgitation, pulmonary hypertension, and previous MI, predicted MACE as well. Moreover, LM/3-vessel CAD, atherosclerotic risk factors burden (≥3 risk factors), and nonsinus rhythm emerged as relevant predictors of MACE too. Univariate hazard ratios with 95% confidence intervals of all conventional variables are shown in Table 2.

Multivariate analysis confirmed the prognostic relevance of LVEF (3.0 [1.7−5.2]; p<0.001) and identified only LM/3−vessel CAD (2.0 [1.4−2.8]; p<0.001) and smoking (1.7 [1.1−2.5]; p<0.01) as independent predictors of MACE. The inclusion of all conventional variables significant at univariate analysis improved the model predictability compared to the LVEF alone (*χ*^2^ 46 versus 21, F-test: p<0.001).

### 3.2. Prognostic Role of CMR 

#### 3.2.1. All-Cause Mortality

CMR metrics of LV volume, ejection fraction, and mass as well as total burden of LGE were strongly associated with all-cause mortality (LVEDV 6.7 [3.1−14.4]; LVEF 5.4 [3.0−9.6]; LV mass 3.5 [1.4−8.7]; LGE 7.6 [3.0−19.2]). However, an abnormal SPCMR result was not a predictor of death (1.1 [0.5−2.2]). Hazard ratios of CMR univariate analysis versus all-cause mortality are presented in Table 2.

Multivariate analysis, which introduced CMR at the last step, showed that CMR variables retain a prognostic value once LVEF at echocardiography and all other significant conventional variables have been taken into account. Indeed, CMR assessment slightly improved the model fit (*χ*^2^ 100 versus 95, F-test p<0.05) with respect to conventional variables alone. The presence of large amount of LGE, namely, replacement of more than 40 percent LV myocardium (equal to the 95^th^ percentile of the entire population), was the sole independent prognostic indicator among CMR metrics (3.4 [1.3−8.8]; p<0.05). Independent prognostic factors were also a reduced LVEF at echocardiography (4.2 [2.0−9.0]; p<0.001), the occurrence of an ACS in the follow-up (6.7 [2.9−15.8]; p<0.001), and an increased LV mass (2.9 [1.3−6.6]; p<0.01). Hazard ratios of the final multivariate model versus all-cause mortality are summarized in Table 3(a).

#### 3.2.2. Major Adverse Cardiovascular Events

CMR parameters were strongly associated with MACE in terms of LV dimensions (LVEDV 3.0 [1.6−5.8]), LV function (LVEF 2.6 [1.7−4.0]), fibrosis (LGE 5.1 [2.7−9.4]), and stress-induced perfusion abnormalities (2.3 [1.6−3.5]). Hazard ratios of CMR univariate analysis versus MACE are presented in Table 2.

After correction for the effect of conventional variables, introduction of CMR variables into the multivariate analysis significantly improved the model fit (*χ*^2^ 83 versus 62, F-test p<0.001). Furthermore, LGE and stress-induced perfusion abnormalities were the best predictors of the composite endpoint (3.3 [1.7–6.3] and 2.1 [1.4−3.2], respectively). The only others significant factors were LM/3-vessel CAD (1.9 [1.4−2.8]), LVEF (2.5 [1.4−4.4]), and smoking (1.5 [1.0−2.2]). Hazard ratios the final multivariate model versus MACE are shown in Table 3(b).

## 4. Discussion

Our study aimed at assessing prognostic power of CMR in a contemporary population with stable CAD and optimal medical treatment, in which CMR was used on top of standard conventional risk assessment. Main findings of the study were as follows: (1) new evidence in the long term of prognostic relevance of LGE as an independent predictor of all-cause mortality; (2) lack of independent prognostic value of SPCMR versus all-cause mortality; (3) confirmation of independent prognostic value of a comprehensive CMR exam, including stress perfusion assessment, for the prediction of a composite endpoint of morbidity and mortality.

At the time of enrollment closure the study had 465 patients that were followed up for a median time of 5.2 years. We primarily investigated the hard endpoint of all-cause mortality. Fifty deaths were observed in the follow-up period, corresponding to a global mortality rate of 10.8% and an annualized event rate of 2.1%. For comparison, all-cause mortality in clinical trials assessing different treatment strategies in stable CAD was in the range 1.3-2.7% [4–8].

### 4.1. Prediction of All-Cause Mortality: LGE

Quantification of fibrosis with LGE was confirmed to be independently associated with mortality. Replacement of large amount of myocardium with scar, namely, of more than 40 percent of LV mass, carried a mean 3.4-fold increase of risk of death after correction for all other factors, in particular LVEF. Given the length of the follow-up of our study, this finding is a confirmation in the long term of what has emerged in recent years from a series of studies showing a negative prognostic significance of myocardium replacement by fibrotic scar, beyond its effect on contractility [11–13].

### 4.2. Prediction of All-Cause Mortality: Stress-Induced Ischemia

Exploring predictors of all-cause mortality, we unexpectedly found that stress-induced perfusion abnormalities at CMR are not independently correlated with prognosis. This finding was quite unpredicted if we consider (1) the good sensitivity and specificity shown by SPCMR for ischemia detection, also in comparison with established imaging techniques like single photon emission tomography [10]; (2) the results of a recent publication demonstrating the utility of SPCMR to reclassify patient risk beyond standard clinical variables (in particular those at moderate/high pretest risk) [14]; (3) numerous publications, summarized in recent meta-analyses [9, 15], showing a significant prognostic value of SPCMR.

In accordance with the literature, patients with a normal SPCMR study have a 1-year mortality of less than 1%, a level of risk significantly inferior to that of patients with positive stress testing. Consequently, the reason why stress perfusion data miss their prognostic significance when SPCMR is used on top of a conventional risk stratification process, as the present study seems to suggest, is not easily understandable. In detail, we found that a significant myocardial ischemia (involving>10% of LV, in accordance with recent guidelines) is not useful to predict mortality once all other well-known significant variables from the clinical history, electrocardiogram, and echocardiography, in particular LVEF, have been considered.

Over the last few decades, significant changes occurred in medical therapy of patients with CAD and atherosclerosis in general, due to the marketing of new drugs like statins, ACE-inhibitors/ARB or thienopyridine, and the wider use of old but efficacious drugs like aspirin. Consistent results of large randomized clinical trials showing reduction of hard events [16–18] have made the use of these drugs mandatory in patients with signs of atherosclerosis. This evidence together with factors like public health policies that reduce smoking has been advocated to explain changes recently observed in atherosclerosis biology and epidemiology: (i) significant decline over time of large atheromas and increase of plaques with more fibrous, noninflammatory characteristics in biobanked carotid plaques [19], (ii) shift in the presentation pattern of ACS with declining of ST segment elevation and rising non-ST segment elevation myocardial infarction incidence [20], and (iii) accumulating evidences that coronary artery bypass grafting and percutaneous coronary intervention may reduce composite endpoints but lack convincing data of an effect on global mortality in stable CAD [4–8], warranting new large international research studies like the ISCHEMIA study [21].

An optimized pharmacologic treatment is methodologically important to minimize the confounding effect of a suboptimal treatment. This goal was achieved in the population we studied thanks to a general policy of guidelines implementation adopted by our department. Compared to the aforementioned clinical trials, the population we investigated had similar levels of treatment with statins (76% versus 73-95%), ASA (85% versus 80-96%), and ACE-inhibitors/ARB (79% versus 30-92%).

Bearing in mind these considerations, the lack of independent prognostic relevance of stress-induced perfusion abnormalities versus mortality, shown by the present study, is not totally surprising. Indeed, optimal medical treatment of the cohort we studied might have hampered the prognostic impact of myocardial perfusion abnormalities, for example, by modifying atherosclerotic plaques stability. Conversely, lower levels of adherence to medical treatment in the study by Shah et al. (statins 50%, ASA 52% and ACE-inhibitors/ARB 44%) might have emphasized the relevance of stress-induced perfusion abnormalities, driving different conclusions about independent prognostic value of SPCMR. Moreover, differences in baseline characteristics between our study and previous studies, for example, higher prevalence of patients with known CAD or MI (86% and 64% in our cohort, respectively), might have influenced predictive value of ischemia versus mortality.

In the present study an indirect confirmation of the low relevance of inducible ischemia in predicting mortality may be considered the lack of independent prognostic value of incident revascularization procedures (49% of patients with positive SPCMR and 24% of the entire cohort underwent revascularization during the follow-up) despite a protective effect emerged at univariate tests. Moreover, none of ischemia related factors we examined, namely, CAD extension, presence of ST segment depression at electrocardiogram, and overall burden of atherosclerotic risk factors, emerged as relevant variables.

### 4.3. Prediction of MACE

**Figure 3:**
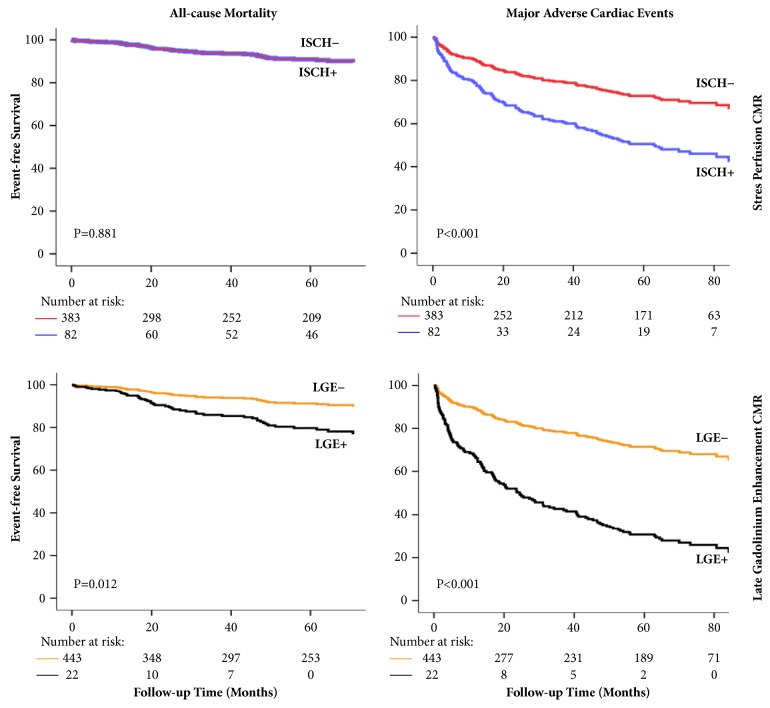
*Independent prognostic value of stress perfusion defects and late gadolinium enhancement at CMR*. Cox proportional model all-cause mortality-free (left panels) and MACE-free (right panels) survival curves, adjusted for all other significant variables. They show that the presence of ischemia in more than 10% of LV mass (ISCH+) is not independently associated with all-cause mortality whereas it predicts the occurrence of MACE (upper panels). Conversely, the presence of scar in more than 40% of LV mass (LGE+) is independently associated with both all-cause mortality and MACE (lower panel). Follow-up period is truncated to 100 months. P value is derived with the log-rank test. MACE = major adverse cardiac event; CMR = cardiovascular magnetic resonance.

The prognostic impact of CMR on a composite endpoint of mortality and relevant morbidities, such as hospitalization for new onset heart failure or ACS and myocardial revascularization procedures unrelated to CMR exam, was confirmed in the present study. CMR introduction into multivariate analysis significantly improved the model fit (p<0.001). Notably, in the final model, LGE and stress-induced perfusion abnormalities were the best predictors of MACE, performing better than LVEF. Large scar at LGE and significant perfusion abnormalities on SPCMR carried a mean 3.3- and 2.1-fold increase of risk of MACE after the correction for all other significant variables. These data confirm the results of previous studies showing that SPCMR is a powerful tool to predict future cardiovascular events [14, 22, 23]. Moreover, they highlight that CMR prognostic value is additional to a careful conventional assessment. Mortality-free and MACE-free survival curves of SPCMR and LGE adjusted for all other significant variables are depicted in Figure 3.

### 4.4. Limitations of the Study

We intentionally defined relatively loose, “real-world”, entry criteria to enroll a population as representative as possible of referral of a standard outpatient CAD cardiology clinic, bearing in mind that the results of the randomized clinical trials are often difficult to translate into clinical practice due to the stringency of their enrollment criteria. However, loose selection criteria might have hampered the prognostic value of stress-induced ischemia at CMR in specific subsets of patients with stable CAD.

This is a single centre observational study that needs to be confirmed by a randomized multicentre study before drawing definitive conclusions about SPCMR role as a stratifying tool in contemporary population with stable CAD.

Female sex was underrepresented in the study population. Accordingly, some caution must be kept in the inference of the results of the study to female patients.

Patients were enrolled in the study for a relatively long period of time. Although the study protocol, in particular CMR protocol, remained unchanged over time, this might be a source of bias.

## 5. Conclusions

Approaching contemporary populations with clinically stable CAD that already receives an optimal evidence based medical treatment: (i) myocardial viability investigation with LGE can be considered a useful tool to further stratify the risk of death in the long term beyond a careful standard clinical and echocardiography assessment; (ii) accurate investigation of myocardial ischemia through SPCMR evaluation does not seem to independently predict mortality; (iii) a comprehensive CMR assessment, including a SPCMR, may be a useful facility to predict morbidity as well as mortality and thus to select subgroups of patients at high risk and high absorption of economical and medical resources.

## Figures and Tables

**Table 1:** Baseline characteristics and differences between patients without and with primary outcome. (all-cause mortality).

	**All patients**	**Event free**	**With events**	**P Value** **∗**
	**(n=465)**	**(n=415)**	** (n=50)**	
ANTHROPOMETRY				
Age (years)	63 ± 11	63 ± 11	67 ± 10	0.006
Male sex	372 (80%)	334 (81%)	38 (76%)	0.454
Body mass index (kg/m2)	26 ± 4	26 ± 4	26 ± 5	0.088
CAD RISK FACTORS				
Family history of CAD	201 (43%)	183 (44%)	18 (38%)	0.454
Smoking	283 (61%)	245 (59%)	38 (76%)	0.018
Diabetes	88 (19%)	75 (18%)	13 (26%)	0.170
Hypertension	267 (57%)	238 (57%)	29 (57%)	0.417
Hypercholesterolemia	271 (58%)	241 (58%)	30 (60%)	0.763
No. of CV risk factors	2.4 ± 1.2	2.4 ± 1.2	2.6 ± 1.0	0.358
CLINIC HISTORY				
Previous CAD diagnosis	398 (86%)	350 (84%)	48 (96%)	0.027
Previous myocardial infarction	298 (64%)	257 (62%)	41 (82%)	0.005
LM or 3-vessel CAD	165 (35%)	144 (35%)	21 (42%)	0.292
NYHA classification (III class)	15 (3%)	11 (3%)	4 (8%)	0.111
Revascularization in the follow-up	112 (24%)	106 (26%)	6 (12%)	0.032
PHARMACOLOGICAL THERAPY				
*β*-blockers	361 (78%)	321 (78%)	40 (80%)	0.692
Ca^++-^antagonist	97 (21%)	85 (21%)	12 (24%)	0.563
Nitrates	184 (40%)	161 (39%)	23 (46%)	0.325
Loop diuretics	146 (31%)	122 (29%)	24 (48%)	0.007
Aldosterone antagonist	54 (12%)	44 (11%)	10 (20%)	0.050
ACE-inhibitors/ARB	369 (79%)	330 (80%)	39 (78%)	0.802
ASA	396 (85%)	351 (85%)	45 (90%)	0.308
Statins	355 (76%)	317 (76%)	38 (76%)	0.952
Anticoagulant use	28 (6%)	22 (5%)	6 (12%)	0.117
ECG				
Heart rate (bpm)	64 ± 12	64 ± 11	70 ± 14	<0.001
Non sinus rhythm	14 (3%)	11 (3%)	3 (6%)	0.317
QRS duration (msec)	104 ± 19	103 ± 18	111 ± 24	0.021
QTc interval (msec)	423 ± 31	421 ± 31	436 ± 33	0.002
LV hypertrophy	66 (14%)	57 (14%)	9 (18%)	0.440
LBBB	55 (12%)	43 (10%)	12 (24%)	0.005
RBBB	59 (13%)	51 (12%)	8 (16%)	0.486
ST segment depression	28 (6%)	24 (6%)	4 (8%)	0.758
Negative T waves	217 (47%)	186 (45%)	31 (62%)	0.021
Q waves	178 (38%)	158 (38%)	20 (41%)	0.660
ECHOCARDIOGRAPHY				
LVEDV (ml/m^2^)	60 ± 30	58 ± 29	72 ± 29	0.006
LVESV (ml/m^2^)	29 ± 17	27 ± 15	44 ± 26	<0.001
LVEF (%)	53 ± 13	54 ± 12	43 ± 14	<0.001
LVWMSI	1.4 ± 0.4	1.3 ± 0.4	1.7 ± 0.5	<0.001
LV mass (g)	197 ± 64	195 ± 61	220 ± 86	0.053
LV diastolic dysfunction (≥pseudo-normal)	42 (9%)	34 (8%)	8 (16%)	0.119
Mitral regurgitation (≥ moderate)	65 (14%)	51 (12%)	14 (27%)	0.006
Pulmonary hypertension (sPAP>35 mmHg)	34 (7%)	27 (8%)	7 (14%)	0.102
RVIT dilatation (>40 mm)	30 (7%)	27 (7%)	3 (6%)	1.000
RV dysfunction (TAPSE<15 mm)	35 (8%)	28 (7%)	7 (14%)	0.122
CARDIAC MAGNETIC RESONANCE				
CMR LVEF (%)	54 ± 15	56 ± 14	43 ± 18	<0.001
CMR LV mass (g)	153 ± 40	151 ± 37	174 ± 55	0.006
CMR LVEDV (ml/m^2^)	70 ± 46	69 ± 47	85 ± 37	0.014
CMR LGE (% of LV mass)	11 ± 13	10 ± 12	19 ± 18	<0.001
CMR myocardial stress induced perfusion abnormality	82 (18%)	73 (18%)	9 (18%)	0.943

CAD = coronary artery disease; CV = cardiovascular; LM = left main; NYHA = New York heart association; ACE = angiotensin converting enzyme; ARB = angiotensin receptor blocker; ASA = acetylsalicylic acid; QTc = corrected QT; LBBB = left bundle branch block; RBBB = right bundle branch block; LVEDV = left ventricle end diastolic volume, LVESV = left ventricle end systolic volume; LVEF = left ventricle ejection fraction; LVWMSI = left ventricle wall motion score index; TAPSE = tricuspid annular plane systolic excursion; RVIT = right ventricle inflow tract; CMR = cardiac magnetic resonance; LGE = late gadolinium enhancement.

*∗*Pearson *χ*^2^ or Fisher's exact test for categorical data; Student's *t*-test or Mann–Whitney for continuous data.

**Table 2:** Univariate Cox analysis of conventional assessment and CMR metrics for all-cause mortality and MACE.

	**All-Cause Mortality**		**MACE**	
	Hazard Ratio (95%CI)	*P* Value	Hazard Ratio (95%CI)	*P* Value
ANTHROPOMETRIC				
Age (≥75 years)	2.6 (1.4−4.9)	0.003	1.6 (1.0−2.4)	0.036
Male sex	1.1 (0.8−1.5)	0.577	1.0 (0.8−1.2)	0.674
Body mass index (>30)	1.5 (0.8−3.0)	0.244	1.1 (0.7−1.8)	0.668
RISK FACTORS				
Family history of CAD	0.8 (0.4−1.4)	0.391	0.9 (0.6−1.3)	0.609
Smoking (previous or active)	2.2 (1.2−3.5)	0.017	1.7 (1.2−2.3)	0.005
Diabetes	1.4 (1.2−4.2)	0.206	1.5 (1.0−2.1)	0.056
Hypertension	0.8 (0.5−1.4)	0.488	1.1 (0.8−1.6)	0.448
Hypercholesterolemia	1.1 (0.6−2.2)	0.714	1.2 (0.9−1.8)	0.258
No. of CV risk factors (≥3)	1.5 (0.8−2.6)	0.168	1.6 (1.1−2.2)	0.013
CLINIC				
Previous CAD diagnosis	4,2 (1.0−17.3)	0.046	2.4 (1.3−4.6)	0.007
Previous myocardial infarction	2.7 (1.3−5.6)	0.006	1.2 (0.8−1.7)	0.392
LM or 3-vessel CAD	1.5 (0.8−2.6)	0.166	2.3 (1.6−3.2)	<0.001
NYHA classification (≥III)	3.3 (1.2−9.2)	0.022	2.5 (1.2−5.4)	0.018
Revascularization in the follow-up	0.4 (0.2−0.9)	0.037	-	
ACS in the follow-up	4.9 (2.2−10.8)	<0.001	-	
THERAPY				
*β*-blockers	1.2 (0.6−2.3)	0.536	1.0 (0.7−1.5)	0.941
Ca^++-^antagonist	1.2 (0.6−2.3)	0.536	1.1 (0.7−1.7)	0.556
Nitrates	1.3 (0.7−2.2)	0.380	1.1 (0.8−1.6)	0.473
Loop diuretics	2.2 (1.3−3.9)	0.005	1.5 (1.1−2.2)	0.021
Aldosterone antagonist	2.4 (1.2−4.8)	0.013	1.4 (0.9−2.3)	0.158
ACE-inhibitors/ARB	0.9 (0.5−1.8)	0.841	1.3 (0.8−2.0)	0.297
ASA	1.7 (0.7−4.3)	0.262	1.3 (0.8−2.2)	0.253
Statins	1.0 (0.5−2.0)	0.943	1.0 (0.6−1.4)	0.831
Anticoagulant	2.3 (1.0−5.5)	0.053	1.9 (1.1−4.4)	0.027
ECG				
Heart rate (>75 bpm)	2.3 (1.2−4.3)	0.011	1.7 (1.1−2.6)	0.018
Non sinus rhythm	2.8 (0.9−9.1)	0.082	2.2 (1.0−4.8)	0.038
QRS duration (>120 msec)	3.4 (1.9−6.3)	<0.001	2.1 (1.4−3.3)	<0.001
QTc interval (≥460 msec)	3.3 (1.8−6.2)	<0.001	1.6 (1.0−2.6)	0.035
LV hypertrophy	1.4 (0.7−2.8)	0.380	1.2 (0.7−1.9)	0.483
LBBB	2.3 (1.2−4.4)	0.013	1.7 (1.0−2.7)	0.032
RBBB	1.4 (0.6−2.9)	0.431	1.4 (0.9−2.2)	0.161
ST segment depression	1.4 (0.5−3.8)	0.537	1.7 (0.9−3.2)	0.089
Negative T waves	1.9 (1.1−3.4)	0.028	1.4 (1.0−1.8)	0.048
Q waves	1.1 (0.6−1.9)	0.774	1.3 (1.0−2.0)	0.101
ECHOCARDIOGRAPHY				
LVEDV (≥105 ml/m^2^)§	3.6 (1.7−7.7)	0.001	1.4 (0.7−2.7)	0.370
LVESV (≥75 ml/m^2^)§	9.4 (4.4−20.2)	<0.001	2.4 (1.2−4.9)	0.016
LVEF (≤30%)	8.0 (4.0−16.0)	<0.001	3.2 (1.9−5.5)	<0.001
LVWMSI (≥2.32)§	5.5 (2.2−13.9)	<0.001	3.3 (1.7−6.5)	0.001
LV mass (≥310 g)§	4.4 (2.0−9.4)	<0.001	2.0 (1.1−3.6)	0.025
LV diastolic dysfunction (≥pseudo-normal)†	2.3 (1.1−4.8)	0.035	1.8 (1.1−3.0)	0.025
Mitral regurgitation (≥moderate)‡	2.5 (1.3−4.7)	0.005	1.5 (0.9−2.3)	0.098
Pulmonary hypertension (sPAP>35 mmHg)	2.3 (1.0−5.1)	0.040	1.7 (1.0−2.8)	0.065
RVIT dilatation (>40 mm)	1.0 (0.3−3.2)	0.989	1.1 (0.5 – 2.2)	0.892
RV dysfunction (TAPSE<15 mm)	2.2 (1.0−4.9)	0.053	1.1 (0.6−2.1)	0.706
CMR				
CMR LVEDV (≥122 ml/m^2^)§	6.7 (3.1−14.4)	<0.001	3.0 (1.6−5.8)	0.001
CMR LVEF (<35%)	5.4 (3.0−9.6)	<0.001	2.6 (1.7−4.0)	<0.001
CMR LV mass (≥236 g)§	3.5 (1.4−8.7)	0.008	1.4 (0.6−3.5)	0.443
CMR LGE (>40%)§	7.6 (3.0−19.2)	<0.001	5.1 (2.7−9.4)	<0.001
CMR myocardial stress induced perfusion abnormality	1.1 (0.5−2.2)	0.881	2.3 (1.6 – 3.5)	<0.001

CMR = cardiovascular magnetic resonance; MACE = major adverse cardiac events; CAD = coronary artery disease; CV = cardiovascular; LM= left main; NYHA =New York heart association; ACS = acute coronary syndrome; ACE =angiotensin converting enzyme; ARB = angiotensin receptor blocker; ASA =acetylsalicylic acid; LBBB = left bundle branch block; RBBB = right bundle branch block; LVEDV = left ventricular end diastolic volume; LVESV = left ventricular end systolic volume; LVEF = left ventricular ejection fraction; LVWMSI = left ventricular wall motion score index; LV = left ventricle/ventricular; sPAP = systolic pulmonary artery pressure; RVIT = right ventricular inflow tract; RV = right ventricle; TAPSE = tricuspid annular plane systolic excursion; LGE = late gadolinium enhancement.

† based on trans-mitral diastolic flow and pulmonary vein flow evaluation.

‡ based on effective regurgitant orifice area.

§ cut-off equal to the 95% percentile of the entire population.

**Table:** (a) Final model of Cox multivariate analysis for all-cause mortality

	Hazard Ratio	95% CI	*P* value
ACS in the follow-up	6.7	2.9−15.8	<0.001
LVEF on echocardiography (≤30%)	4.2	2.0−9.0	<0.001
QTc interval (≥460 msec)	2.8	1.5−5.4	0.002
LV mass (≥220 g)	2.9	1.3−6.6	0.009
TotalLGE burden (≥ 40% LV mass)	3.4	1.3−8.8	0.012
Heart rate (>75 bpm)	2.0	1.0−3.8	0.041

**Table:** (b) Final model of Cox multivariate analysis for MACE

	Hazard Ratio	95% CI	*P* value
Total LGE burden (>40% LV mass)	3.3	1.7−6.3	<0.001
CMR myocardial stress induced perfusion abnormality	2.1	1.4−3.2	<0.001
LM or 3−vessel CAD	1.9	1.4−2.8	<0.001
LVEF on echocardiography (≤30%)	2.5	1.4−4.4	0.002
Smoking	1.5	1.0−2.2	0.038

ACS = acute coronary syndrome; LV = left ventricle; LVEF = left ventricle ejection fraction; QTc = corrected QT interval; LGE = late gadolinium enhancement; CMR = cardiovascular magnetic resonance; MACE = major adverse cardiac events; LM = left main coronary artery; CAD = coronary artery disease.

## Data Availability

The data used to support the findings of this study are available from the corresponding author upon request.

## References

[1] Horton R. (2012). GBD 2010: understanding disease, injury, and risk. *The Lancet*.

[2] Cortigiani L., Picano E., Coletta C. (2001). Safety, feasibility, and prognostic implications of pharmacologic stress echocardiography in 1482 patients evaluated in an ambulatory setting. *American Heart Journal*.

[3] Hachamovitch R., Berman D. S., Shaw L. J. (1998). Incremental prognostic value of myocardial perfusion single photon emission computed tomography for the prediction of cardiac death: differential stratification for risk of cardiac death and myocardial infarction. *Circulation*.

[4] Henderson R. A., Pocock S. J., Clayton T. C. (2003). Seven-year outcome in the RITA-2 trial: Coronary angioplasty versus medical therapy. *Journal of the American College of Cardiology*.

[5] Boden W., O’Rourke R., Teo K. (2007). Optimal medical therapy with or without PCI for stable coronary disease. *The New England Journal of Medicine*.

[6] BARI 2D Study Group, Frye R. L., August P., Brooks M. M. (2009). A randomized trial of therapies for type 2 diabetes and coronary artery disease. *The New England Journal of Medicine*.

[7] Hueb W., Lopes N., Gersh B. J. (2010). Ten-year follow-up survival of the medicine, angioplasty, or Surgery Study (MASS II): A randomized controlled clinical trial of 3 therapeutic strategies for multivessel coronary artery disease. *Circulation*.

[8] Stergiopoulos K., Brown D. L. (2012). Initial coronary stent implantation with medical therapy vs medical therapy alone for stable coronary artery disease: Meta-analysis of randomized controlled trials. *JAMA Internal Medicine*.

[9] Lipinski M. J., McVey C. M., Berger J. S., Kramer C. M., Salerno M. (2013). Prognostic value of stress cardiac magnetic resonance imaging in patients with known or suspected coronary artery disease: a systematic review and meta-analysis. *Journal of the American College of Cardiology*.

[10] Schwitter J., Arai A. E. (2011). Assessment of cardiac ischaemia and viability: Role of cardiovascular magnetic resonance. *European Heart Journal*.

[11] Catalano O., Moro G., Perotti M. (2012). Late gadolinium enhancement by cardiovascular magnetic resonance is complementary to left ventricle ejection fraction in predicting prognosis of patients with stable coronary artery disease. *Journal of Cardiovascular Magnetic Resonance*.

[12] Kelle S., Roes S. D., Klein C. (2009). Prognostic Value of Myocardial Infarct Size and Contractile Reserve Using Magnetic Resonance Imaging. *Journal of the American College of Cardiology*.

[13] Roes S. D., Kelle S., Kaandorp T. A. M. (2007). Comparison of Myocardial Infarct Size Assessed With Contrast-Enhanced Magnetic Resonance Imaging and Left Ventricular Function and Volumes to Predict Mortality in Patients With Healed Myocardial Infarction. *American Journal of Cardiology*.

[14] Shah R., Heydari B., Coelho-Filho O. (2013). Stress cardiac magnetic resonance imaging provides effective cardiac risk reclassification in patients with known or suspected stable coronary artery disease. *Circulation*.

[15] Sozzi F. B., Iacuzio L., Civaia F. (2015). Incremental value of normal adenosine perfusion cardiac magnetic resonance: Long-term outcome. *American Heart Journal*.

[16] Baigent C., Keech A., Kearney P. M., Cholesterol Treatment Trialists' (CTT) Collaborators (2005). Efficacy and safety of cholesterol-lowering treatment: prospective meta-analysis of data from 90,056 participants in 14 randomised trials of statins. *The Lancet*.

[17] Al-Mallah M. H., Tleyjeh I. M., Abdel-Latif A. A., Weaver W. D. (2006). Angiotensin-Converting Enzyme Inhibitors in Coronary Artery Disease and Preserved Left Ventricular Systolic Function. A Systematic Review and Meta-Analysis of Randomized Controlled Trials. *Journal of the American College of Cardiology*.

[18] Pilgrim T., Windecker S. (2014). Antiplatelet therapy for secondary prevention of coronary artery disease. *Heart*.

[19] Van Lammeren G. W., Den Ruijter H. M., Vrijenhoek J. E. P. (2014). Time-dependent changes in atherosclerotic plaque composition in patients undergoing carotid surgery. *Circulation*.

[20] Katz J. N., Shah B. R., Volz E. M. (2010). Evolution of the coronary care unit: Clinical characteristics and temporal trends in healthcare delivery and outcomes. *Critical Care Medicine*.

[21] The ISCHEMIA Study. Accessed 22 Nov 2016. https://www.ischemiatrial.org

[22] Bodi V., Sanchis J., Lopez-Lereu M. P. (2007). Prognostic value of dipyridamole stress cardiovascular magnetic resonance imaging in patients with known or suspected coronary artery disease. *Journal of the American College of Cardiology*.

[23] Bingham S. E., Hachamovitch R. (2011). Incremental prognostic significance of combined cardiac magnetic resonance imaging, adenosine stress perfusion, delayed enhancement, and left ventricular function over preimaging information for the prediction of adverse events. *Circulation*.

